# Dengue Virus Serotype 4, Northeastern Peru, 2008

**DOI:** 10.3201/eid1511.090663

**Published:** 2009-11

**Authors:** Brett M. Forshey, Amy C. Morrison, Cristhopher Cruz, Claudio Rocha, Stalin Vilcarromero, Carolina Guevara, Daria E. Camacho, Araceli Alava, César Madrid, Luis Beingolea, Víctor Suarez, Guillermo Comach, Tadeusz J. Kochel

**Affiliations:** US Naval Medical Research Center Detachment, Lima and Iquitos, Peru (B.M. Forshey, A.C. Morrison, C. Cruz, C. Rocha, S. Vilcarromero, C. Guevara, T.J. Kochel); University of California, Davis, California, USA (A.C. Morrison); Laboratorio Regional de Diagnostico e Investigación del Dengue y otras Enfermedades Virales, Maracay, Estado Aragua, Venezuela (D.E. Camacho, G. Comach); Instituto Nacional de Higiene y Medicina Tropical "Leopoldo Izquieta Pérez", Guayaquil, Ecuador (A. Alava); Naval Hospital, Guayaquil (C. Madrid); Dirección General de Epidemiología, Ministerio de Salud, Lima (L. Beingolea); Instituto Nacional de Salud, Lima (V. Suarez)

**Keywords:** Dengue virus, Peru, South America, vector-borne infections, mosquitoes, viruses, dispatch

## Abstract

In 2008, dengue virus serotype 4 (DENV-4) emerged in northeastern Peru, causing a large outbreak and displacing DENV-3, which had predominated for the previous 6 years. Phylogenetic analysis of 2008 and 2009 isolates support their inclusion into DENV-4 genotype II, forming a lineage distinct from strains that had previously circulated in the region.

Infection by any 1 of 4 distinct dengue virus serotypes (DENV-1 through DENV-4) can result in disease manifestations ranging from asymptomatic or mild to severe outcomes, including dengue hemorrhagic fever (DHF) and dengue shock syndrome. Several lines of evidence point toward secondary infection by a heterologous serotype as one of the critical risk factors for DHF (*1*), underscoring the need to monitor circulating DENV serotypes and genotypes.

In Latin America, >30 countries and regions have reported DENV circulation, totaling >900,000 dengue fever cases, 26,000 DHF cases, and 300 deaths in 2007 (*2*). Following the breakdown of a hemisphere-wide *Aedes aegypti* mosquito eradication campaign conducted in the mid-20th century, vector populations expanded and all 4 DENV serotypes reemerged or were reintroduced into the Western Hemisphere. Outbreaks of DENV-2 and DENV-3 were first detected in the 1960s, followed by the introduction of DENV-1 in 1977 and DENV-4 in 1981 (*3**,**4*).

Since its introduction into the Americas in 1981, DENV-4 has circulated continuously in the Caribbean basin (*5*) and northern South America with little evidence of widespread transmission further south into the continent during the past 25 years (*4**,**6**,**7*). We report the emergence of DENV-4 strains belonging to genotype II in the tropical rainforest and coastal regions of northern Peru, replacing DENV-3 subtype III (*8*) as the predominant strain in the region.

## The Study

**Figure 1:**
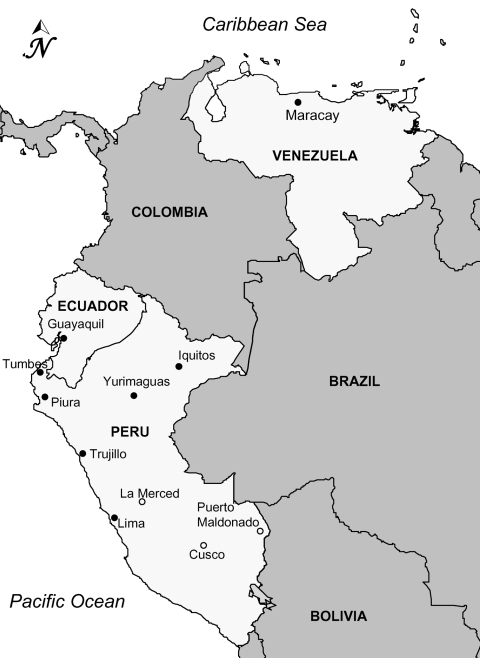
Map of study sites in Ecuador, Peru, and Venezuela. The study in Peru and Ecuador was operated jointly by the US Naval Medical Research Center Detachment with each country’s Institutes of Health. Samples from Venezuela were collected in and around Maracay through the Aragua State Epidemiologic Surveillance Program from patients with suspected dengue virus (DENV) infection. Cities denoted by filled circles indicate study sites where DENV serotype 4 strains were isolated, whereas open circles denote active study sites where no DENV–4 circulation was detected during the course of the study.

Patients with acute, undifferentiated, febrile illness were recruited into a clinic-based surveillance study conducted jointly by the US Naval Medical Research Center Detachment (NMRCD) and the Peruvian and Ecuadorian Institutes of Health (Figure 1). Study protocols (NMRCD.2000.0006 [Peru] and NMRCD.2001.0002 [Ecuador]) were approved by the Naval Medical Research Center Institutional Review Boards in compliance with all US federal regulations governing the protection of human subjects.

Patient sera were injected onto African green monkey Vero cells or *Ae. albopictus* C6/36 cells and examined for a range of arboviruses, including all 4 DENV serotypes, by immunofluorescent assay. From 2000 through 2008, DENV-3 (1,572 isolates) was the dominant serotype in circulation in the study sites, followed by DENV-1 (205 isolates) and DENV-2 (87 isolates). From the initiation of the study in May 2000 until February 2006, DENV-4 circulation was rarely detected in the NMRCD-affiliated study sites in either country; the exceptions were a small number of isolates in 2000 in Tumbes, Peru (n = 2) and Guyaquil, Ecuador (n = 6).

Low-level DENV-4 transmission was again detected in Ecuador and coastal Peru during 2006 and 2007, in isolates from patients in Tumbes, Trujillo, and Guayaquil, none of whom had reported recent history of travel outside their respective areas. DENV-4 continued to circulate in sites along the northern coast of Peru in 2008 and 2009, in co-circulation with DENV-1. In February 2008, DENV-4 spread to the cities of Iquitos and Yurimaguas, located in the Loreto Department in the tropical rainforest region of northeastern Peru. By October 2008, DENV-4 had nearly completely displaced DENV-3, which had been the only serotype detected in the region during the previous 3 years. Nine (56%) of 16 DENV isolates obtained from febrile patients in August 2008, 55 (85%) of 65 isolates obtained in September 2008, and 305 (98%) of 311 isolates obtained from October 2008 through February 2009 were DENV-4. After the introduction of DENV-4, the total number of DENV-3 isolates decreased during peak months of DENV transmission (October through February) from 176 during 2006–2007 and 420 during 2007–2008 to <10 isolates during the same period in 2008–2009. More recently (March 2009), DENV-4 strains have spread south to Lima, the capital city of Peru, causing a small, localized outbreak on a military base.

To characterize the DENV-4 isolates, a 1,485-bp sequence covering the entire mature envelope (E) gene was amplified and sequenced from a representative set of viruses from Guayaquil (n = 6), Tumbes (n = 6), Piura (n = 2), Trujillo (n = 1), Iquitos (n = 9), Yurimaguas (n = 7), and Lima (n = 2), all collected during 2000 through 2009. Isolates collected in Peru since 2006 shared >99.5% nucleotide identity but exhibited <97% identity with DENV-4 strains collected in Ecuador and coastal Peru in 2000. At the amino acid level, the 2006–2009 isolates were nearly invariant, with <1 amino acid difference.

**Figure 2:**
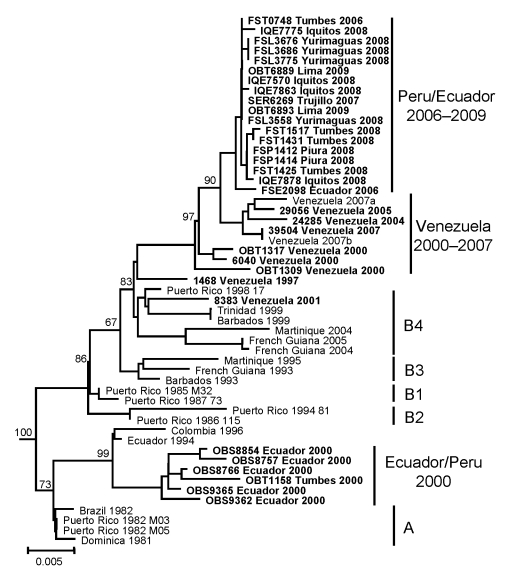
Phylogenetic analysis of the envelope gene of dengue virus serotype 4 (DENV–4) strains from Ecuador, Peru, and Venezuela. Similar topologies were observed from neighbor-joining (depicted), maximum likelihood, and maximum parsimony analyses, implemented in PAUP* v.4.0b10 (*12*). The general time reversible model of evolution was used for neighbor-joining and maximum-likelihood analyses. DENV-4 genotype I strains (not shown) were included as an outgroup. Bootstrap values (based on 1,000 replicates) >65 are shown at major nodes. Isolates first reported in this study are shown in **boldface** and with sample identification code. Some sequenced isolates from Peru and Venezuela that share high nucleotide identity (>99.7%) with depicted strains were omitted to reduce redundancy and improve clarity of the figure. Sequences were deposited in the GenBank database under the accession nos. GQ139572–GQ139577 (Ecuador), GQ139547–GQ139571 (Peru), and GQ139578–GQ139591 (Venezuela). Scale bar indicates number of nucleotide substitutions per site.

For further characterization, the DENV-4 strains from Ecuador and Peru were compared with DENV-4 sequences from Latin America (*9**,**10*) and Southeast Asia (*11*) available from the GenBank database. In addition, to provide a wider array of recent isolates from the Caribbean region, DENV-4 isolated from febrile patients in Aragua State, Venezuela (n = 15) collected during 2000 through 2007 were sequenced. Based on phylogenetic analyses, all DENV-4 isolates belong to genotype II (data not shown), although the 2006–2009 isolates segregated into a markedly different clade than the strains from 2000 (Figure 2). The 2000 isolates clustered more closely with a previous 1994 Ecuador isolate (*9*), related to the initial 1981 Caribbean introduction DENV-4 strains (designated as subtype A). The 2006–2009 isolates were most closely related to recent DENV-4 isolates from Venezuela, with as low as 0.8% nucleotide divergence, and formed a lineage distinct from previously published DENV-4 Caribbean basin strains (*9**,**10*), with strong bootstrap support (Figure 2). This lineage is distinguished from previously reported DENV-4 genotype II strains by 3 conserved amino acid variations in the E protein: S64L, A235T, and S403A.

## Conclusions

We report DENV-4 expanding rapidly through northern Peru, particularly in the Loreto Department in the tropical rainforest region, spreading through a population immunologically naïve to this serotype. In the past 2 decades, populations in northern Peru have been exposed to the other 3 DENV serotypes, thus increasing the possibility for severe disease, including DHF. In Iquitos, DHF was first reported during a DENV-3 epidemic in 2004 (M. Sihuincha and C. Rocha, pers. comm.). DHF had not been observed after the introduction of DENV-2 despite large numbers of infected residents (*13*), presumably due to cross-protection afforded by prior infection with DENV-1 (*14*). For the currently circulating lineage of DENV-4, the levels of either cross-protection or antibody-dependent enhancement of infection resulting from prior heterologous infection remain to be determined.

The mechanisms responsible for DENV-3 displacement in Loreto are unclear. Following several years of DENV-3 circulation in the region, serotype-specific antibody prevalence is high (≈45% of the population, based on virus neutralization assays [T.J. Kochel, unpub. data]). However, it is unlikely that herd immunity is sufficient to explain the dramatic decrease in DENV-3 transmission. The presence of broadly cross-protective antibodies during the acute and early convalescent phases following DENV-4 infection, when combined with the large number of DENV-3 immune persons, could suppress transmission of DENV-3 strains (*15*). Another possibility is serotype competition within the vector species. Analysis of virus strains from *Ae. aegypti* mosquitoes collected during the transitional period could help elucidate whether such intertypic competition is occurring.

Genetically, the 2006–2009 isolates analyzed in this study do not appear to be related to viruses collected from Ecuador and northern Peru during 2000, which were similar to the initial Latin American introduction strains (Figure 2). Instead the 2006–2009 isolates were most closely related to viruses from Venezuela collected during 2000 through 2007, forming a lineage distinct from the DENV-4 genotype II B4 lineage (*10*). Genetic conservation among isolates from Peru and similarity with isolates from Venezuela support an introduction event into northern Peru and Ecuador from the northern region of South America before 2006, and a subsequent introduction from coastal Peru into Loreto, although more data from other regions of South America and the Caribbean basin would be necessary to more clearly delineate the geographic spread of this virus strain.
