# Erratum—Vol. 15, No. 11

**DOI:** 10.3201/eid1601.ER1601

**Published:** 2010-01

Two numbers were listed incorrectly in the article Dengue Virus Serotype 4, Northeastern Peru, 2008 (B.M. Forshey et al.). The final sentence before the Conclusions should read: “This lineage is distinguished from previously reported DENV-4 genotype II strains by 3 conserved amino acid variations in the E protein: S64L, A222T, and S354A.” The article has been corrected online (www.cdc.gov/eid/content/15/11/1815.htm).

